# *Zelus renardii* Roaming in Southern Italy

**DOI:** 10.3390/insects13020158

**Published:** 2022-01-31

**Authors:** Nada Lahbib, Ugo Picciotti, Valdete Sefa, Sonia Boukhris-Bouhachem, Francesco Porcelli, Francesca Garganese

**Affiliations:** 1 Dipartimento di Scienze del Suolo, della Pianta e degli Alimenti, University of Bari Aldo Moro, 70126 Bari, Italy; nadalahbib48@gmail.com (N.L.); valdetesefa@gmail.com (V.S.); francesco.porcelli@uniba.it (F.P.); francesca.garganese@uniba.it (F.G.); 2 Faculty of Sciences of Tunis, University of Tunis El-Manar, Rommana, Tunis 1068, Tunisia; 3 Laboratory of Plant Protection, INRAT—National Institute of Agronomic Research of Tunisia, Carthage University, Ariana 2049, Tunisia; bouhachems@gmail.com; 4 Laboratory of Phytopathology, Department of Marine Science and Applied Biology, University of Alicante, San Vicente del Raspeig, 03690 Alicante, Spain; 5 CIHEAM—Centre International de Hautes Etudes Agronomiques Méditerranéennes, Mediterranean Agronomic Institute of Bari, 70010 Valenzano, Italy

**Keywords:** alien invasive or quarantine, stenophagous predator, infection and vector biocontrol, Harpactorinae, reference metadata analysis

## Abstract

**Simple Summary:**

The leafhopper assassin bug *Zelus renardii* (Hemiptera: Reduviidae) first entered Europe in 2012 and has since acclimatised. *Z. renardii* is now a common insect predator in agroecosystems and urban and peri-urban areas. We performed a metadata analysis of 165 years of *Z. renardii* literature. Moreover, we provide laboratory tests of *Z. renardii* predation. The latter indicates *Z. renardii* interplay with relevant insect targets, such as *Philaenus spumarius*, *Neophilaenus* *campestris, Bactrocera oleae*, *Kermes vermilio*, *Nidularia pulvinata*, *Harmonia axyridis*, *Apis mellifera*, *Aleurocanthus spiniferus*, *Aleurothrixus floccosus, Macrohomotoma gladiata*, *Drosophila suzukii*, *Drosophila melanogaster*, *Megaselia scalaris,* Pseudococcidae, Miridae, and Issidae. Furthermore, predation on Aphrophoridae and other olive pests brands *Z. renardii* as a good mass-rearing candidate for inundative biocontrol programs of *Xylella fastidiosa pauca* ST53 infections and could also reduce damage caused by other olive pests. Overall, this reduviid is harmless to humans and beneficial insects.

**Abstract:**

This study collects data from the literature and updates our *Zelus renardii* Kolenati, 1856 (Leafhopper Assassin Bug, LAB) prey knowledge. The literature consists of ca. 170 entries encompassing the years 1856 to 2021. This reduviid originated in the Nearctic region, but has entered and acclimatised in many Mediterranean countries. Our quantitative predation experiments—in the laboratory on caged plants plus field or environmental observations—confirm that LAB prefers a selected array of prey. Laboratory predation tests on living targets (Hemiptera, Coleoptera, Diptera, and Hymenoptera) agree with the literature. *Zelus renardii* prefers comparatively large, highly mobile, and readily available prey. LAB preferences on available hemipterans targets suggest that *Zelus renardii* is a good inundative biocontrol agent for *Xylella fastidiosa* *pauca* ST53 infections. LAB also prey on other important olive pests, such as *Bactrocera oleae*. Therefore, *Zelus renardii* is a major integrated pest management (IPM) component to limit *Xylella fastidiosa* pandemics and other pest invasions.

## 1. Introduction

Studies on *Macrohomotoma gladiata* (Kuwayama, 1908) [1] and *Aleurocanthus spiniferus* (Quaintance, 1903) in Apulia [2] disclosed the acclimation of the Leafhopper Assassin Bug (LAB) or *Zelus renardii* Kolenati, 1856 (Reduviidae: Harpactorinae—Zr) in southern Italian areas threatened by *Xylella fastidiosa* (Wells, Raju et al., 1986—Xf) *pauca* ST53 invasion [3,4].

The first observations of the LAB attacking and killing abilities versus *Philaenus spumarius* (L., 1758—meadow spittlebug) [5,6] and *Neophilaenus campestris* (Fallén, 1805) suggest LAB as a biocontrol agent of Xf vectors. *Philaenus spumarius* is the primary Xf vector in Italy with a 25–71% transmission efficiency [7,8]. *Philaenus spumarius* causes much of the olive (*Olea europaea* L., 1753) infections and the invasive spread of Xf over territories [9].

Expansion of olive orchards under organic practices requires new crop protection strategies to manage the transmission by Xf vectors [10,11]. LAB’s bionomics could fit the needs of organic olive pest control by using the mass-reared reduviid as a biocontrol agent against Xf vectors and other pests. However, this proposal has been met with various opinions and concerns.

LAB is considered a generalist predator [12,13,14,15,16,17]. However, even generalist predators show prey preferences that satisfy their developmental and reproductive needs [18]. The range of prey depends on physiological, physical, and behavioural limitations [19]. Accordingly, the prey list of LABs in the field usually has fewer species than those reported in the literature, as for other generalist predators [18,19,20,21].

This contribution contains two sections: the first deals with available LAB references and their content; the second includes experimental data from laboratory tests, describing LAB’s actions versus relevant prey.

We aim to share the results of the *Z. renardii* tests carried out in Italy. These tests evaluate if LAB uses Italian prey vicariant of the North American ones or beneficial insects as targets such as honeybees. The data will help clarify LAB’s role as a potential biocontrol agent of Xf vectors and infection.

## 2. Materials and Methods

### 2.1. LAB References Retrieval

**Figure 1:**
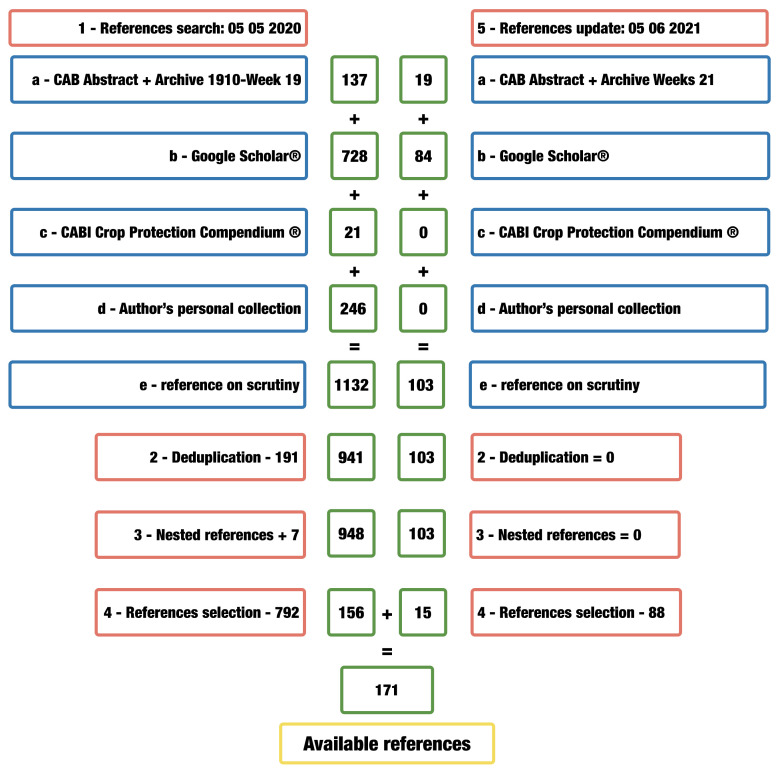
The process adopted to select LAB references analysis, used in this study.

We retrieved LAB references from several repositories, using the systematic review method (Figure 1). The bibliography was consulted in two steps using an exact string, namely “*Zelus renardii*”. The first step is an initial search on databases for the 1856–2020 interval; the second step is to search for bibliographic updates published in 2021, one year later, in the same databases. Databases selected were CAB-Abstract via Ovid, Crop Protection Compendium^®^ (CPC) and Google Scholar. The period from 1856 to 2021 was analysed.

A further nested search into each publication’s references list gathered some additional relevant articles that were included in this study.

### 2.2. LAB Field Collection

Sweeping nets, beating trays, and direct plant observations served to collect LAB adults, nymphs, and egg batches. Table 1 shows LAB collection places with GPS coordinates, plants, and insects associated with *Zelus* findings. The LABs for this study originate from collections that began in August 2014 and ended in December 2019. Images (in .raw file format) illustrate the collection site and context. We registered all data collected in a FileMaker^®^ database (version Pro 12, Claris International Inc, Cupertino, CA, USA).

### 2.3. Laboratory Work

Our tests aim to understand the LAB predatory attitude versus Palearctic vicariant prey in Italy. These tests compare the use of the referenced *Zelus*-sympatric prey in North America with those vicariant in Italy.

LAB rearing and tests were performed in our laboratory, between 18 and 25 °C and enlightened by side windows, apart from background artificial ring lighting during the Aphrophoridae and honeybees’ tests.

Table 2 summarises rearing arenas and performed tests, revealing the LAB broad behaviour and efficacy versus eleven different prey species of different instars, living either motile or immotile, on their host plants or other backgrounds. Laboratory rearing or testing depended on insect availability for continuous living prey supply.

We used LABs of both sexes to test prey acceptance, apart from the trial on *Bactrocera oleae* Gmelin, 1790, since only predator males were available at the time in the laboratory. In the predation tests, LAB and potential prey had 10 min to interact, if not reported otherwise. When interactions lasted longer than 10 min, tests were left to continue until the interplay between LAB and prey remained.

### 2.4. LAB Rearing

**Figure 2:**
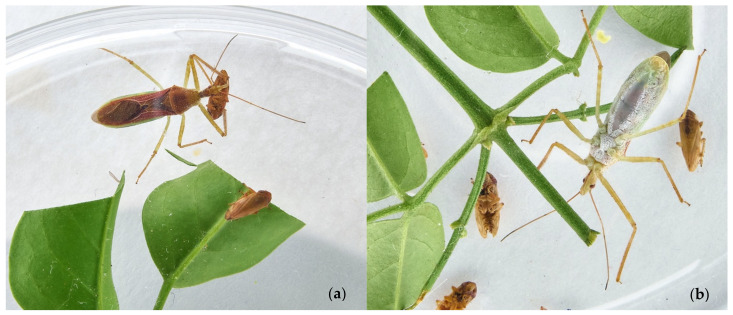
(**a**,**b**) LAB female reared in a Petri dish on jasmine twigs with meadow spittlebugs available prey.

*Zelus* were reared continuously in the laboratory in Petri dishes (Figure 2a,b) of various sizes (Table 2). Vented polystyrene rearing flasks also hosted these reduviids, depending on their instar. Crystal-clear methacrylate or high-density polyethene also provided excellent observation opportunities. Pieces of water-soaked paper or cotton wool maintained the proper environmental RH% (Relative Humidity), and a filter paper disk floor offered an appropriate substrate for the insects. Only Italian *Zelus* was reared in the laboratory. *Zelus* collected abroad were immediately killed and preserved in 80% EtOH (Ethanol).

**Figure 3:**
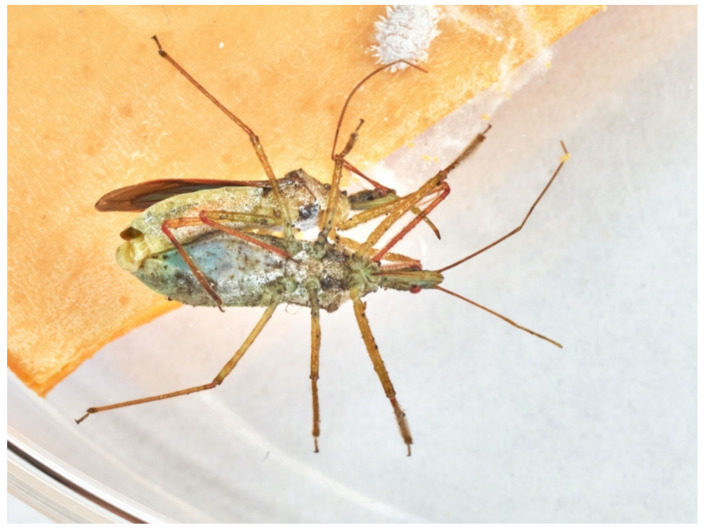
LABs mating in the laboratory.

*Zelus*, paired into a 9 cm ∅ Petri dish (Figure 3), later returned to individual rearing plates. Newly born naiades were moved to new containers to give them adequate space. Non-single use rearing arenas were cleaned and sanitised with 70% EtOH every 48 h to reduce the risk of contamination.

### 2.5. LAB Rearing on Drosophila melanogaster, Drosophila suzukii, and Megaselia scalaris

*Drosophila melanogaster* L., 1758, was grown in 4 L plexiglass flasks on modified [11] meridic artificial substrate [22]. *Drosophila suzukii* (Matsumura, 1931) was grown on ripened and overripened fruits available in the season. *Drosophila suzukii* is an alien invasive pest in Italian vineyards [23,24], which has recently acclimatised to Apulia [25]. *Megaselia scalaris* (Loew, 1866) was reared on espresso coffee wet waste capsules placed in an opaque wastebasket.

### 2.6. LAB Rearing on Macrohomotoma gladiata

Fresh watered *Ficus* sp. twigs in polystyrene-vented rearing flasks (Table 2) harbouring active *M. gladiata* adults and nymphs hosted single wild adult *Zelus*. Psylla was paired and bred in flasks with *Zelus*. The twigs maintained proper moisture and supported both predator and prey. The psyllid *M. gladiata* is an alien invasive pest in the EPPO region [26,27].

### 2.7. LAB Test on Pseudococcidae

*Planococcus citri* (Risso, 1813) and *Planococcus ficus* (Signoret, 1875) were reared on butternut squashes (*Cucurbita moschata* (Duchesne) Duchesne ex Poir., 1818) [28,29], in CIHEAM-IAMB facilities or collected on grapes. We placed small mealybugs groups into Petri dishes (Table 2).

### 2.8. LAB Test on Aleyrodidae

Fresh *Citrus* sp. twigs (40 cm long and with 15–20 leaves each) infested by *Aleurocanthus spiniferus* (Quaintance, 1903) and *Aleurothrixus floccosus* (Maskell, 1896)—underside the leaves—were placed in polystyrene-vented rearing flasks (Table 2).

### 2.9. LAB Test on Coccomorpha

Fresh *Q. ilex* twigs (30 cm long, with 20–30 leaves each) infested by *Kermes vermilio* Planchon, 1864, and *Nidularia pulvinata* (Planchon, 1864) served to test LAB predation. *O. europaea* twigs (20 cm long, with 30–35 leaves each) infested by *Saissetia oleae* (Olivier, 1791) hosted LAB predation tests on Coccidae. These tests were performed in polystyrene-vented rearing flasks (Table 2).

### 2.10. LAB Test on Xylella vectors, Miridae and Issidae

Aphrophoridae, Miridae and Issidae were captured using sweeping conifers by nets in 5 min rounds. A large funnel was used to transfer hemipteran into a 5 L PET-vented plastic tank. Avoiding the use of aspirators prevented a de-pressuring shock and the consequent unpairing of the prey. The sweeping also gathered twigs of *Cupressus sempervirens* L., 1753, *Cupressus arizonica* Greene, 1882, and *Thuja occidentalis* L., 1753 food plants. These were kept for moisture and fed to the prey. Each test used only 15, fully active Hemiptera adults, to ensure no attempts to escape from the tank.

### 2.11. LAB Test on Harmonia axyridis

Twenty-three *Harmonia axyridis* (Pallas, 1773) were collected on *Citrus* sp. infested by whiteflies (*A. spiniferus* and *A. floccosus*) in the University of Bari Aldo Moro Campus tested with LAB. Each ladybird interplayed in a Petri dish (Table 2) with one LAB.

### 2.12. LAB Test on Bactrocera oleae, the Olive Fly

Eleven LAB adults preyed on 10–12 *B. oleae* during a one-day extended test, intermingled with a day of starvation. The container for the test was a vented parallelepiped polystyrene rearing flask (Table 2). The olive fly puparia were from an oil mill near Molfetta (41.205651° N; 16.573373° E). Adult olive flies escaped from puparia in plexiglass-vented boxes (Table 2) with a plenty food supply [30]. To avoid using teneral adults, the flies hardened in the boxes for a minimum of one day after the escaping and before the tests. We recorded LAB feeding time on each *B. oleae* as we did for the *Xylella* vectors tests.

### 2.13. LAB Test on Apis mellifera

We investigated the ability of *Z. renardii* to prey on *Apis mellifera* L., 1758 (Hymenoptera: Apidae—AM). We placed both insects in Petri dishes (Table 2, 81 cm^3^) on shading support over a LED (4000 K°) ring lamp for 10 min to interact. We used 19 LAB individuals (9 ♀ and 10 ♂) and five mid-aged honeybee workers. Each AM replayed randomly ca. four times. Insect behaviour was recorded (.raw image or full HD movie format) with a digital camera (Olympus^®^ Pen camera, OM Digital Solutions Corporation, Hamburg, Germany). If an interaction lasted more than 10 min, we continued to record until the interplay ended. Scrutinising the interactions resulted in a series of paths of both insects, drawn to follow the tracks taken by LABs and AMs. Path drawings were processed using Adobe Illustrator^®^ (Adobe Inc., San Jose, CA, USA).

### 2.14. LAB and Humans

We reproduced LAB biting events as reported on humans. Ten volunteers placed LABs on the naked skin of the back of their left hand, pushing them progressively and firmly to elicit LAB biting. We recorded the LAB action and marked the piercing point, following its evolution for a week. None of the volunteers had a known history of allergy requiring special care. All LAB died during the tests based on the pressure they had endured.

### 2.15. LAB’s Tests Interpretation Issues

The LAB predation tests here presented did not consider any functional response [31,32]. The tests only supported the data of LAB Nearctic prey during more than a century of studies on various European vicariant prey.

**Figure 4:**
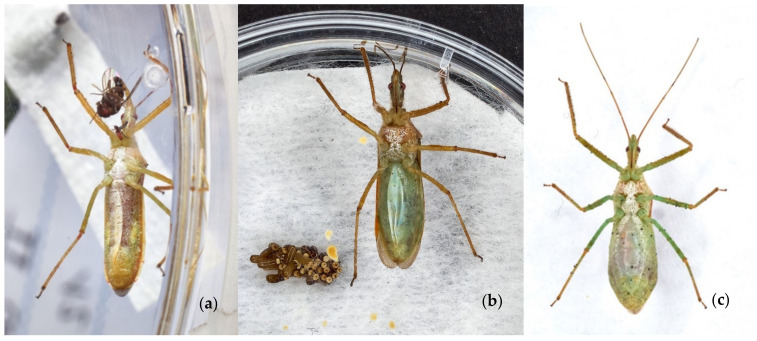
(**a**) starved LAB female displays contracted abdominal fold at the feeding start; (**b**) LAB female with semi-contracted abdominal fold after egg laying, dark-yellow spots are faeces; (**c**) LAB female shows distended abdominal fold after preying.

**Figure 5:**
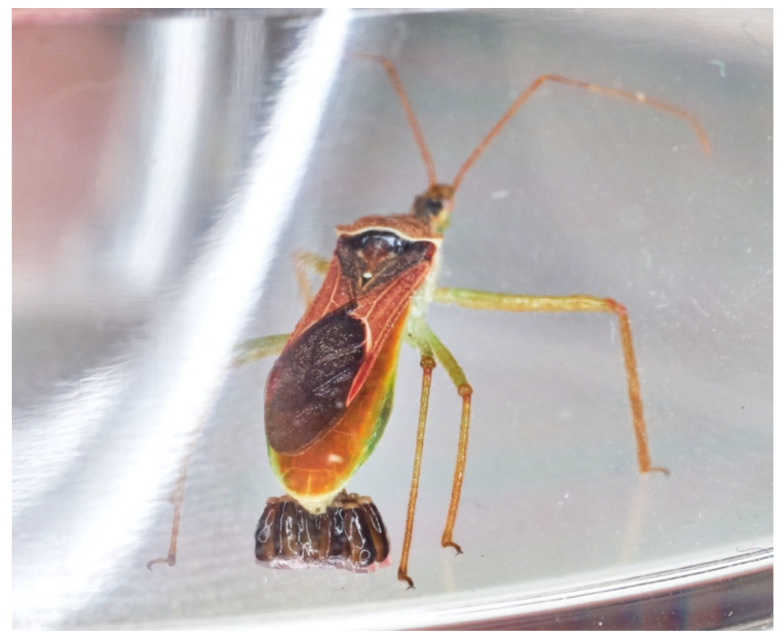
LAB female during egg laying.

The reduviids’ abdominal fold (Figure 4a–c) and defecations indicate feeding after each attack. The status of the abdominal fold allows recognising a starved or engorged individual. Usually, egg laying follows the engorgement of a LAB female (Figure 5).

Post-predation egg laying and hatching of LAB after tests indicate if that particular prey is suitable for predator reproduction. Results clearly state if eggs are missed.

## 3. Results and Discussion

### 3.1. References Retrieval

Literature searches yielded 1235 records. The analysis discarded 191 duplicates plus 880 untraceable documents. Furthermore, seven new articles originated from nested reference scrutiny. Our literature search yielded 171 references for the current study. These references represent 16.27% of the entries retrieved and 13.85% of the total titles (including duplicates) found in the databases.

The references [33,34,35,36,37,38,39,40,41,42,43,44,45,46,47,48,49,50,51,52,53,54,55,56,57,58,59,60,61,62,63,64,65,66,67,68,69,70,71,72,73,74,75,76,77,78,79,80,81,82,83,84,85,86,87,88,89,90,91,92,93,94,95,96,97,98,99,100,101,102,103,104,105,106,107,108,109,110,111,112,113,114,115,116,117,118,119,120,121,122,123,124] gathered information on LAB bionomics and prey preferences (Table S1), ordered per taxon, types of approach, and qualitative or quantitative experience. The literature reports several observations of LAB feeding on various prey, mainly from the Nearctic region. Only a few reports exist for LAB prey from other biogeographical areas.

**Figure 6:**
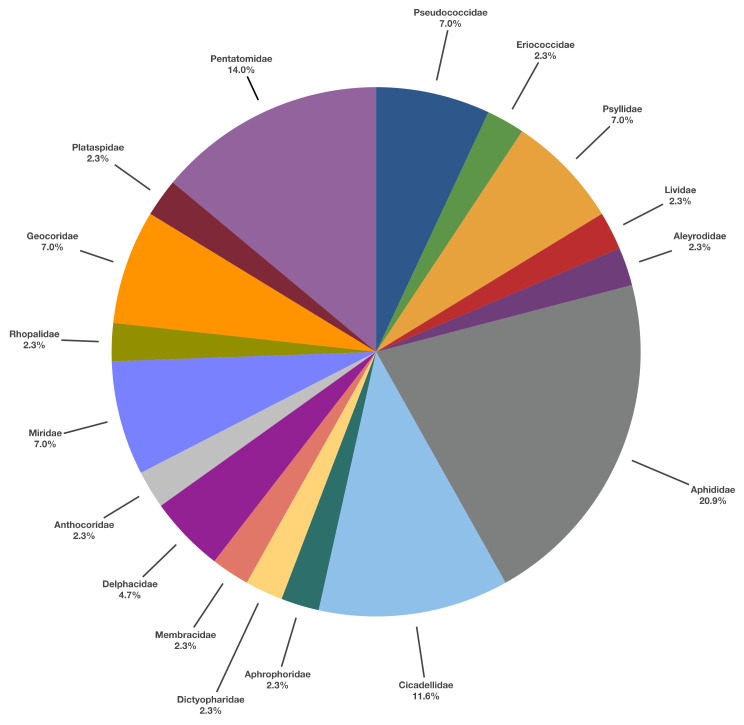
Relevance of Hemiptera families preyed by LAB according to available literature survey.

The literature of LAB prey preferences showed that LAB uses 88 prey species belonging to nine orders. References show ca. 48.9% of the total prey species belong to Hemiptera. Other taxa references—Blattodea and Orthoptera 1.1%, Thysanoptera 4.6%, Neuroptera 5.7%, Lepidoptera 15.9%, Coleoptera 12.5%, Diptera 4.6% and Hymenoptera 5.7%, respectively. The analysis of Hemiptera species references in Figure 6 shows a wide array of prey, with a 20.9% maximum score for Aphididae.

Beneficial insects as *Chrysoperla carnea* (Stephens, 1836), *Adalia bipunctata* (L., 1758) and *A. mellifera* only represent the 2.4%, 1.2% and 1.2%, respectively, of LAB’s predation events under natural conditions, as shown in Table S1. LAB can predate more on them in constricted environments (i.e., cages, boxes, etc.).

Therefore, LAB appears much more stenophagous [19] on Hemiptera than a polyphagous predator. *Zelus longipes* (L., 1767), just as *Z. renardii*, selects prey based on their mobility and size [125].

Literature metadata (Supplementary Material: Table S1) shows that field observations represent 59.5% of total references, the remaining 40.5% being laboratory observations. Reports about LAB prey preferences are qualitative (51.8%) and quantitative (48.2%) in similar percentages. Field experiments report much regarding LAB preference for a certain prey and represent non-quantitative observations, not valid for pest biocontrol forecast and impact evaluation on beneficial arthropods. Furthermore, they do not discuss intra-guilt predation.

*Zelus* is amongst the largest genera of the Reduviidae [126,127,128,129], with ca. 70 described species [33,126,130]. Kolenati [131] described *Zelus renardii* in 1856, and Weirauch [130] accepted the “Leafhopper Assassin Bug” (LAB) as its trivial name. Henry [132] and Zhang [126] revised the genus species placing *Zelus cervicalis* Stål, 1872 as a sister species of *Zelus renardii* but accepting *Zelus peregrinus* Kirkaldy, 1902, *Zelus laevicollis* Champion, 1898, and *Diplodus renardii* Uhler, 1894, as synonyms.

*Zelus renardii* is native of North and Central America [33,42,126,129,130,133,134,135,136,137]. The reduviid widely spread throughout the American continent [42,123,138,139,140,141,142,143,144], Asia and Oceania [143,145,146,147,148,149]. The bug also entered Hawaii [150], and Zimmerman [71] considers *Zelus* a large, mostly tropical American genus.

Several authors [33,151] have reported LAB from mainland Greece, as Simov [152] confirmed. Later, van der Heyden [153] found *Zelus* in Crete. During the same period, *Zelus* entered Spain [133,134], Italy [135,154,155,156], Albania [127], France [157] and Portugal [158]. However, recently, LAB has entered Germany [159], Denmark and the UK via grape transport from Italy and Greece [160]. Today, LAB is considered well established in Europe [136] as well as in Italy [137] and the Iberian Peninsula [161,162,163].

LAB is an alien, acclimated, spreading species in many Mediterranean areas adapted to urban and peri-urban ecosystems [164,165].

Miller [17] considers Reduviidae generalist predators that prey opportunistically on various arthropods, including beneficial insects.

Qualitatively, LAB is a predator of different pests and beneficial insects (Table S1) in the field and laboratory conditions. Quantitatively, its favourite preys belong to the Hemiptera [166] and the Cicadellidae [58], Aphididae [12,13,54] and Psyllidae [1]. Ambrose [16] considers LAB as a “generalist” when introduced and a “specialist” insect pest predator in its habitat. The difficulty is to place this predator in a sharp category, ranging from monophagous specialists to ultra-generalists. However, assigning a particular non-specialised predator to a specific behavioural class (polyphagous, stenophagous, oligophagous) [19] is not immediate due to our limited knowledge of the species diet in nature [12,13,14,15].

LAB is almost an exclusive carnivore [167], differing from many stenophagous and generalist predators that also eat plants and even pests. Mixed-diet insects might reasonably be called generalist omnivores [19]. LAB’s prey choice varies according to ecological conditions such as plant habitat, composition, and abundance of prey species. In the absence of prey, *Z. renardii* naiades could supplement their diet with vegetable-derived *pabula* to survive for short periods [14,168]. Under severe starvation, LAB can turn to cannibalism [1,12,58,82,106], eventually leading to the self-control of the population. LAB is highly adaptive, dispersive, and able to colonise new areas. This may generate density of 50,000–75,000 individuals/ha [105].

LAB prefers mobile prey [58] and does not play an important biological control agent role in cotton fields [169]. LAB presence in orchards and crops depends on prey abundance or prey diversity [105] and mobility. LAB adults show a functional response to prey abundance [100], size [67,106] and the presence of honeydew-producing Hemiptera. The LAB predation strategy depends mainly on prey size [16,130,170,171]. *Zelus* prefers to ambush small prey but to stalk larger ones [105]. A sticky secretion on the anterior tibiae has a decisive role in predation success [137,139,140,170,172,173,174]. This secretion of the forelegs allows sticking the prey on the anterior legs minimising attack failures. Generally, *Zelus* is quite active and voracious with a low attack failure rate. Furthermore, the salivary glands’ enzymes allow ingesting large amounts of prey body mass [175].

LAB lays eggs in batches, each with 12 to 62 units [58]. Each female can lay up to 270 eggs [138] in 2–3 sets during its life [43,112], with a fertility rate ranging from 18 to 64% [43]. The eggs are sub-cylindrical, brown [58,71,122,138,172] and topped by a whitish operculum [58]. Moreover, LAB covers egg masses with a sticky secretion [58,138]. Usually, the reduviid places the egg batches on the upper side of leaves or various substrates. Post-embryonic development consists of five juvenile instars [58,138]. Adults exhibit sexual dimorphism, with bigger females (♀♀ are about 14 mm long and ♂♂ ca. 11 mm) [58,176].

LAB is a predator with a preference for disturbed environments [33,105]. It is a synanthropic [42] insect occurring in urban and peri-urban environments [42,43,114,130,137,161], where it has a greater chance of encountering various prey species.

Human activities facilitate *Z. renardii* passive dispersion, facilitating entrance and exploitation of new countries [130,141,143,145]. The positive correlation between urban environments and reduviid abundance also confirms human-driven LAB passive dispersion.

Force [177] considers r-strategist predators such as *Zelus* less numerically dominant in mature ecosystems and regularly found on a broad geographical or ecological range. Furthermore, r-predators tend to be abundant in perturbed habitats (e.g., agroecosystem or urban areas). They also have a high demographic growth rate being relatively weak competitors. The reduviid life cycle completes in about two months [43,71].

The numerical and functional responses to prey density do not govern a dynamic relationship between the LAB and target prey. Moreover, predators choose specific targets to supplement their amino acid requirements [178].

### 3.2. Field Observations and Tests

LAB synecology and biology feature displayed in Table 3. It is easy to spot LAB hunting in urban and peri-urban areas frequenting hemipteran-infested plants. We collected a total of 126 LABs (Table 3).

LAB collection of wild adults outnumbers the number of eggs and juveniles on plants (Table 3). Assuming a population in equilibrium, the number of eggs and juveniles should be higher than adults [179,180]. The adult population should result in fewer adults than the corresponding eggs and juveniles, contrarily as reported in Table 3. The total number of adults found (64 LABs) is larger than the sum of the eggs, and juvenile stages found (33 and 29, respectively). Adults’ collection is more accessible than pre-imago instars because the individuals are prominent and easier to spot than smaller, slender juveniles. Adults prefer large prey, and require more *pabula* than juveniles. The greater mobility of adults also makes them more visible in the field.

The hemipteran’s honeydew could lure LAB to the plants infested with available prey, causing LAB egg laying and the possible presence of their pre-imagoes. Multiple observations of *Zelus* preying on wandering among *T. alacris*, *O. binotatus*, *A. spiniferus*, *S. coffeae* or *S. oleae* suggest this honeydew luring effect. Fall surveys on the same plants listed in Table 3 did not result in LAB findings. Perhaps LAB adults had moved to overwinter shelters [181]. Further comparative observations on neighbouring clean plants support the hypothesis that LAB is associated with hemipteran honeydews or faeces.

Honeydew-like fluids, or some of the attractive semiochemicals within, could influence *Zelus* behaviour. Honeydew could push the predator to aggregate and persist in a specific place for pest control. The Hemiptera honeydew likely contains volatile organic compounds (VOCs) that may act as kairomones attracting the predator. Other VOCs could originate from honeydew or artificial surrogates or associated microorganisms either drawing antagonists [182,183,184,185] or attracting/repelling pests [186]. These VOCs may participate in behavioural manipulation actions within an IPM strategy to enforce any non-synthetic chemical control action arsenal for bio-management of Xf vectors.

LAB mating lasts about 37 min (Table S2) under laboratory conditions (T = 25 ± 3 °C and RH = 80 ± 5%). Females reject the partner shaking the abdomen [58]. Starved females may try to prey on the approaching male or kill it after the mating. Cannibalism is not a novelty in LAB female rearing, however, male killing is not regular and does not have the sense to replace missing preys.

**Figure 7:**
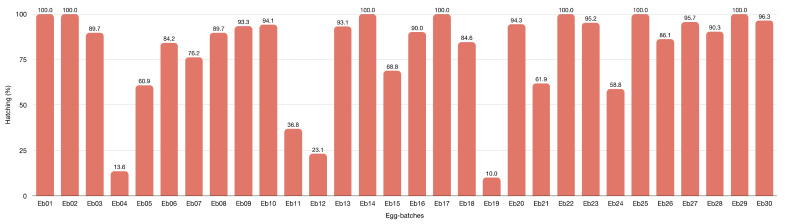
Hatching percentage of thirty egg batches laid after LABs mating, detailed in Table S2. Abbreviations: Eb = egg-batch.

Mbata [112] and Barrera [43] suggest that each LAB female laid 2–3 batches giving an average of 33 eggs with a prolificacy ranging from 18 to 64% [43]. In our rearing, females laid a total of 1112 eggs; females laid an average of three batches averaging 27 eggs (Table S2) [58,138] with a prolificacy of about 80%, with a higher hatching rate (Figure 7) and many new-borns than reported. Moreover, females are polyandrous, and El-Tom [58] considers repeated mating induces more eggs than single-mating. Embryonic development of our LAB lasts 17.4 ± 5.4 days, unlike Swezey [138] and El-Tom [58] that reported LAB embryonic development of about 6–10 days at 25 °C. Our hatching period data is the average value for one-year-long LAB breeding experiments. Swezey [138] and El-Tom [58] report a shorter May–August breeding period. Differences in hatching time may rise from photoperiod, food available or female age. There is a concordance between our breeding data and observations of one-century-long literature.

### 3.3. LAB Rearing on Drosophila melanogaster, Drosophila suzukii and Megaselia scalaris

*Zelus* can be mass reared successfully with a continuous supply of *D. suzukii*, *D. melanogaster* and *M. scalaris*. Interestingly, *D. suzukii* is now another phytophagous species on olive fruits [187]. Therefore, models and joint strategies for bio-management of *Xylella*-vectors (key pest) and secondary olive pests, as *D*. *suzukii,* should consider this LAB attitude.

**Figure 8:**
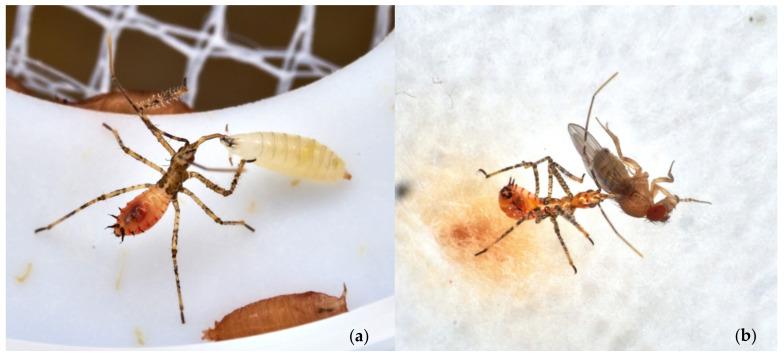
(**a**) LAB naiad feeding on *Drosophila* sp. larva; (**b**) LAB naiad feeding on *D. melanogaster* adult.

The LAB’s juveniles fed on maggots and adults (Figure 8a,b) of both the gnats during the rearing, showing a normal post-embryonic development until adults. Adult reduviids were active, and females laid regularly fertile egg batches that hatched as expected on almost all available surfaces. The egg laying proved that these dipterans are suitable prey for LABs mass rearing.

### 3.4. LAB Rearing on Macrohomotoma gladiata

**Figure 9:**
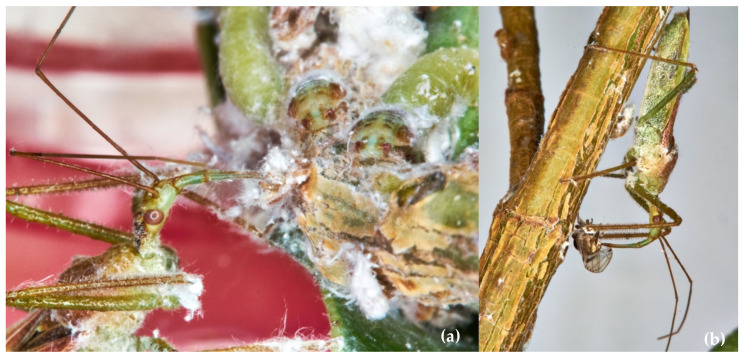
(**a**) LAB female feeding on *Macrohomotoma gladiata* juvenile; (**b**) LAB female feeding on *M. gladiata* adult.

In rearing, each reproductive LAB uses 25–30 *M*. *gladiata* adults per day plus an unknown number of nymphs (Figure 9a,b). *Macrohomotoma gladiata* juveniles are uncountable because sheltered among fig leaves.

*Macrohomotoma gladiata* is a suitable prey for LAB since females reared on them laid weekly viable egg batches with eggs hatching after 10–20 days. If several LABs live in the same cage, *intra*- and frequent *inter*-instar cannibalism occur. Moreover, adults attack and kill nymphs [15] frequently.

### 3.5. LAB Tests on Pseudococcidae

**Figure 10:**
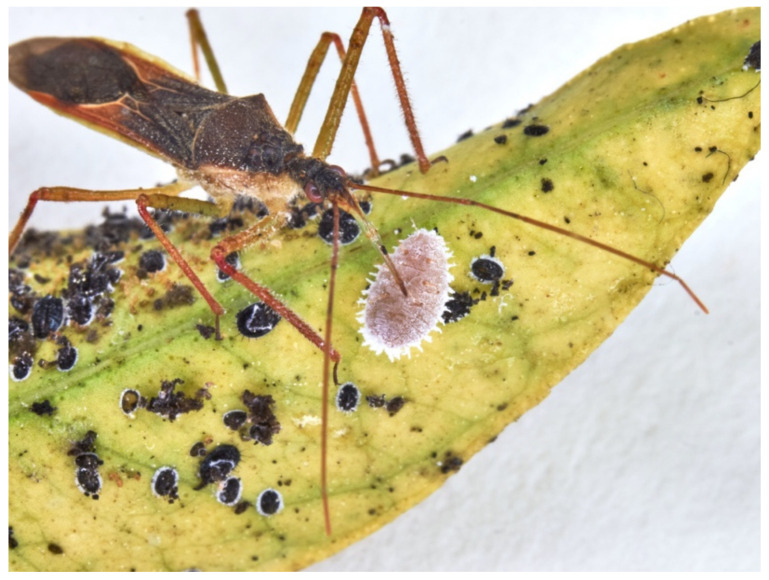
LAB female feeding on *Planococcus citri* adult.

*Zelus*, in general, ignores immotile prey such as Pseudococcidae. Few predations occur when *P. ficus* and *P. citri* move, provoking the immediate reduviid action that spots and pierces them (Figure 10). In this case, feeding lasts 1–2 min. LAB could feed on Pseudococcidae, but it does not lay eggs.

### 3.6. LAB Tests on Aleyrodidae

LAB females survive 7–10 days on mandarin twigs harbouring *A. spiniferus* and *A. floccosus* mixed infestations without laying eggs. *Aleurocanthus spiniferus* is an alien invasive Aleyrodidae, representing the new key pest for *Citrus* spp. orchards in Apulia [188,189]. Few whitefly adults were occasionally preyed upon [51]. Any whiteflies (ca. four individuals for each LAB) died because glued to the predator’s sticky forelegs [174,190], but LAB neither fed on those bodies nor laid eggs.

### 3.7. LAB Tests on Coccomorpha

There is no evidence of LAB predation either on an adult or on crawlers (naiades) of *K. vermilio*, *N. pulvinata* and *S. oleae*. Some crawlers died, stuck to the predator’s glutinous forelegs. No egg laying occurred.

### 3.8. LAB Tests on Xylella vectors, Issidae and Miridae

LAB preys on a plethora of different phytophagous Hemiptera belonging to Aphrophoridae, Issidae, and Miridae. Observations showed that LAB captures its target first, sticking it with the forelegs [174,190]. The insertion of the stylets through the prey intersegmental membranes follows. The saliva injection kills the prey, possibly because the action of proteolytic enzymes (proteinase and endopeptidase) predigest the prey [102]. Afterwards, the predator sucks the lysate obtained from the prey body [102].

**Figure 11:**
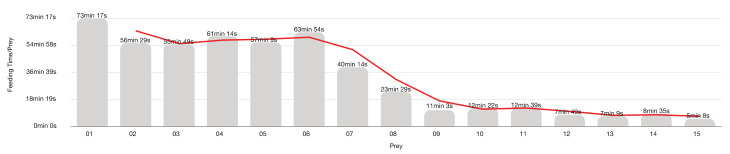
*Z. renardii* average feeding time. Each bar represents the average time that LABs took to feed on each prey.

**Figure 12:**
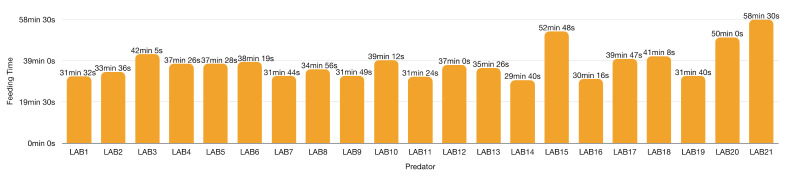
*Zelus renardii* individual average feeding time per 15 hemipterans (Aphrophoridae, Issidae and Miridae) prey, details in Table S3.

Figure 11 and Figure 12 show the feeding time for the 21 LAB (eight females and 13 males) predations experiment on mixed Aphrophoridae, Miridae and Issidae with a maximum of 15 prey per LAB.

*Zelus* preys by ambushing or stalking its prey [105]. LAB predation that occurs within a 60 cm^3^ Falcon flask happens primarily due to accidental encounters between moving predator and prey rather than LAB aggressions. Indeed, the high prey availability creates a great chance of casual prey-predator encounters that prevent LAB from executing its conventional predatory strategies because there is always an available prey around. What we see in Falcon tubes are LABs waiting for the passage of prey to attack and kill it. Prey was so frequent that the LAB stopped feeding on the sixth prey onwards. LABs continued to kill the next prey but did not feed on them.

In Falcon tube experiments, the attack time diminished by the frequencies of the encounters with the prey, and LAB devoted remaining time to feeding only. Thus, the feeding time depends mainly on the prey size and LAB starvation [66,106,125,130,170,171]. LAB takes on average 33 min 5 s to feed on each prey. Overall, predator feeding time decreases with the increasing number of predations. The longer feeding time (73 min 17 s) corresponds to the first prey, and the shorter (5 min 8 s) coincides with the last one.

The trend line shows two time-plateaux splits by an abrupt drop of feeding time, corresponding to the prey from the 7th to the 9th. First plateau—prey 1st to 6th (61 min 19 s)—corresponds to a real feeding time, but the second plateau—prey 10th to 15th (08 min 57 s)—accounts for just killing and carcass abandonment, with any or negligible feeding on prey. The behaviour strongly suggests that engorgement rules the LAB, which does not feed even if it still follows its killing instinct.

LABs show a high predatory aptitude on *Xylella* vectors, killing almost all available Aphrophoridae, Issidae and Miridae. Indeed, LAB laid eggs batches after preying on Aphrophoridae, Issidae and Miridae.

During tests, LAB reused prey carcasses. In the Falcon tubes, LAB can easily be in touch with prey it killed previously. Often the predator drives the stylets in that dead carcass as it does with just killed insects. The behaviour may be relevant in restricted spaces where carcasses are still available. Carcass feeding is possibly negligible in the wild because dead prey abandonment provokes their dispersal in the environment. In small arenas with high prey density, overrepresented carcass feeding occurs. The observation seems a behaviour not previously reported in the literature. The use of such carcasses may help avoid cannibalism in overcrowded LAB mass rearing, eventually.

Differences exist among females and males prey use (on average, ♀♀ 44 min 39 s vs. ♂♂ 33 min 44 s). Apart from sex-related prey exploitation, wild LAB could host not sex-related and non-lethal but weakening viruses, bacteria, apicomplexa, and fungi (yet to be found). LAB collected in the wild has unknown age or sanitary status.

### 3.9. LAB Tests on Harmonia axyridis

**Figure 13:**
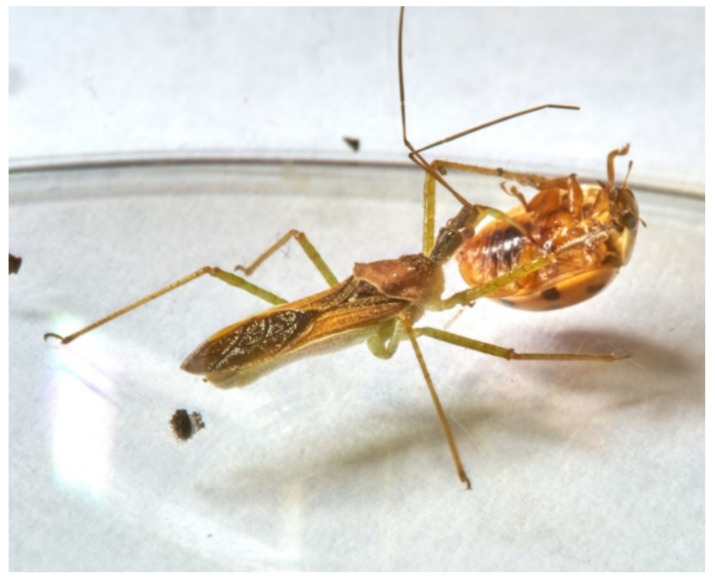
LAB male feeding on *Harmonia axyridis*.

Twenty-three *Harmonia axyridis* underwent attacks in Petri dishes by five LABs (two ♀ and three ♂) that stalked them. Despite the immediate beetle immobilisation after apparent stylet insertion (Figure 13), victims soon recovered from the aggression by walking around; this differs from previous reports [114]. The same LAB also re-attack surviving *H. axyridis* during a subsequent encounter in the Petri dish. We did not test if ladybirds were castrated by the attacks or suffered other significant damages. After this episode, LAB did not release eggs, and the abdominal ventral fold stretched moderately.

### 3.10. LAB Tests on Bactrocera oleae (BO, the Olive Fly)

**Figure 14:**
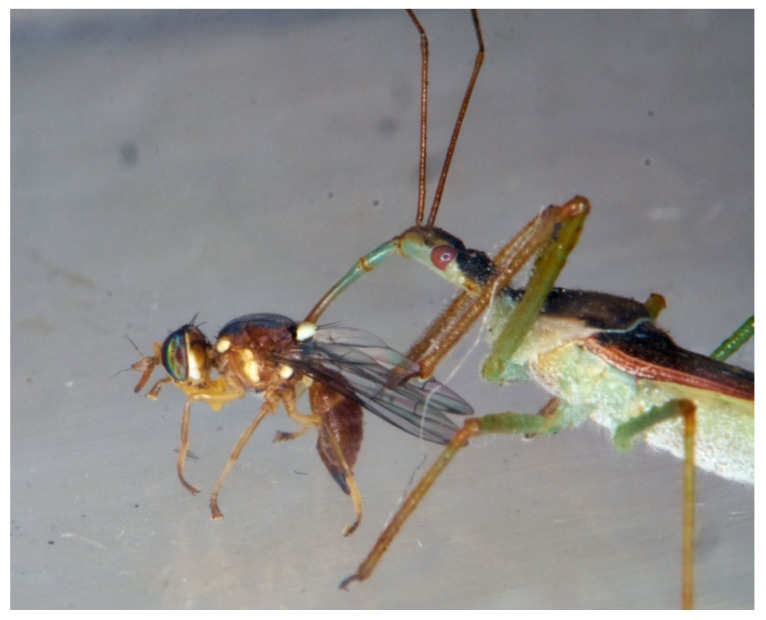
LAB male feeding on *Bactrocera oleae* adult.

LABs attack fruit flies, *Bactrocera cucurbitae* (Coquillett, 1899), in the field [65,113] and thrive on *Ceratitis capitata* (Wiedemann, 1824) and *Anastrepha ludens* (Loew, 1873) [43]. In laboratory tests, LAB males also attack *B. oleae* (Figure 14), killing 10–12 individuals exposed in the arena (Table 2, 580 cm^3^) within 24 h. Feeding time was about 60 min for the first prey but shortened to about 18 min for the 12th. The prey dynamics are consistent with those observed in the Aphrophoridae, Issidae and Miridae tests. Once engorged, LAB reduced the feeding time to the last prey. The remaining BOs disturbed LAB feeding by flying and banging over the reduviid. LABs replied by leaving the feeding prey and killing the BO that irritate them. In total, only five BOs managed to escape from the LAB’s attack.

*Bactrocera oleae* was the key pest of olive trees in southern Italy until evidence of transmission of Xf via Aphrophoridae was formed [4]. From an IPM perspective, predation of BO would allow to manage it as a pest and sustain LAB on the olive orchards as alternative prey to *Xylella* vectors. Indeed, the scarcity or absence of prey would induce the LAB to abandon the olive orchards in search of a new target.

**Figure 15:**
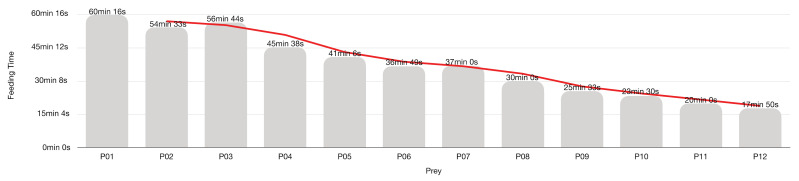
*Zelus renardii* feeding time in minutes recorded in 580 cm^3^ arena each provided 10–12 prey, details in Table S4. Each bar represents the average time that *Z. renardii* took to feed on each prey item.

**Figure 16:**
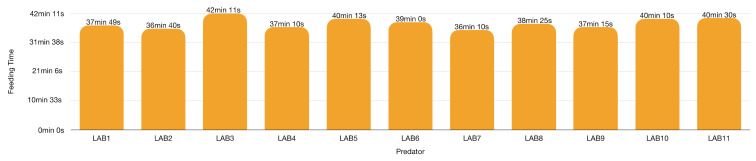
*Zelus renardii* feeding time per prey in minutes recorded in 580 cm^3^ arena per each of the 11 predators, details in Table S4. Each bar represents the average feeding time per 10–12 prey items.

Average feeding times per ambushing predator shows similar lengths, possibly because this test included males only, instead of both males and females as for Aphrophoridae prey (Figure 15). Abdominal ventral fold stretches as LAB feeds on the first prey. LAB takes on average 38 min 36 s to feed on each BO (Figure 16). Males only participated in the test because of vagrants available in winter.

### 3.11. LAB Tests on Apis mellifera (AM)

**Figure 17:**
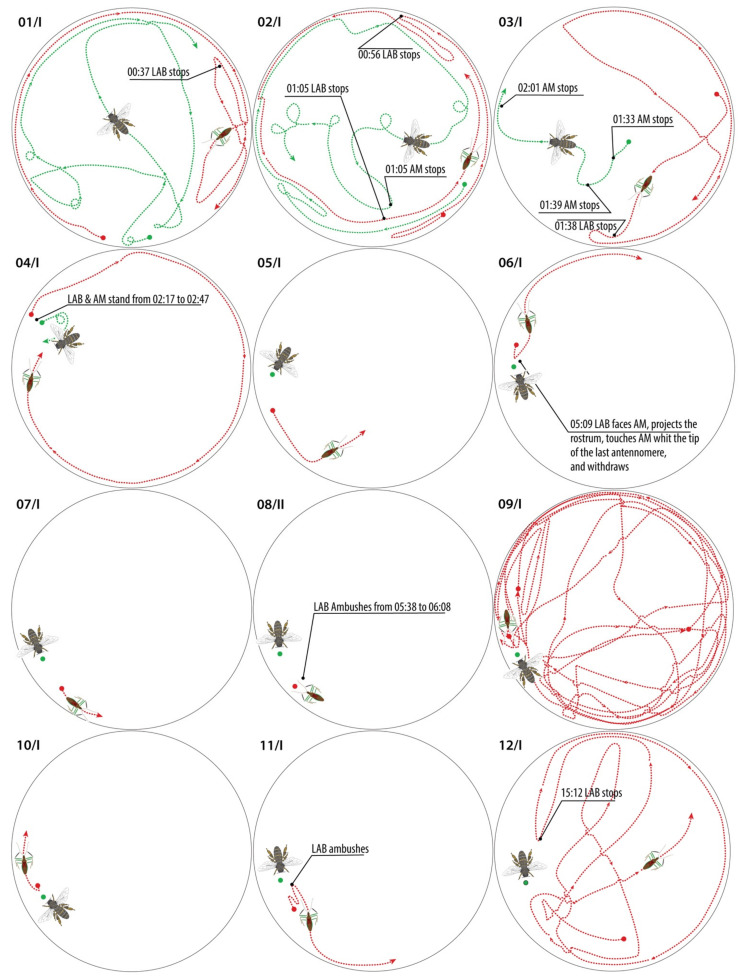

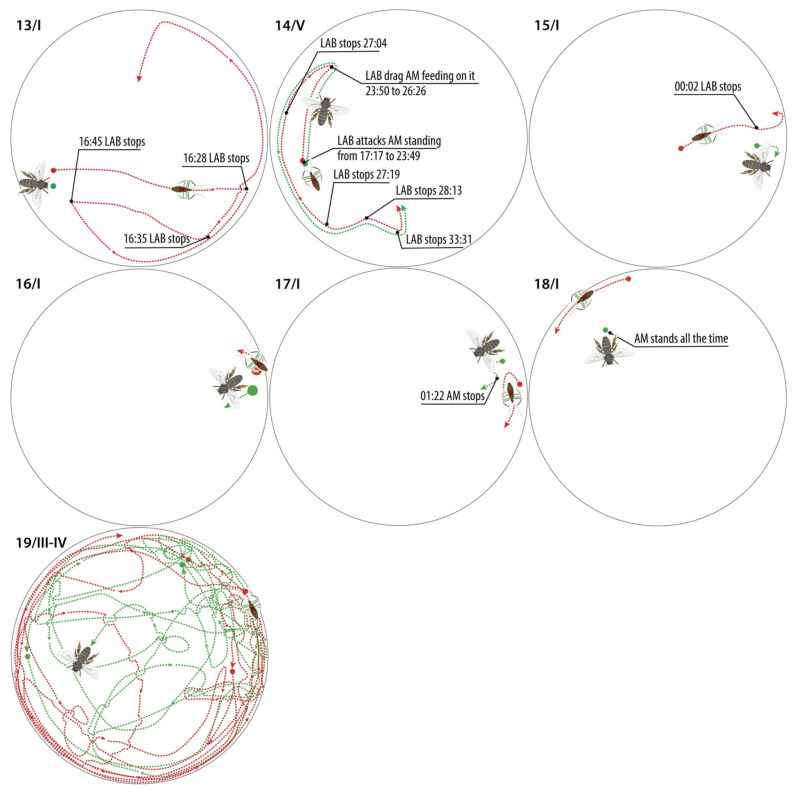
LAB–AM interactions. Green lines are the AM paths, and red lines are the LAB paths. Latin numerals near the scheme describe the behaviours, details in Table S5 and *Zelus*-*Apis* movie (Supplementary Materials).

One of the major concerns against LAB use for inundative releases in the field was the claim about reduviid predation on honeybees. Our experiments with LAB and AM placed together in Petri dishes (Table S5) show five behavioural patterns: (I) reciprocal ignorance, wandering around in the Petri dish; (II) staying almost immotile near or on the release spot; (III) AM walks or flutters versus the escaping LAB; (IV) AM accidentally collide with LAB eliciting its aggressive posture; (V) AM glues on LAB sticking legs that attacks and kills the honeybee defending itself (Figure 17).

Most (73.7%) LAB–AM interactions (Table S5) belong to type I behaviour: reciprocal overlooking with both actors wandering alone in the plate.

In type II behaviour (Table S5), LAB and AM stay motionless, staring at each other at the release point.

Walking across the Petri dish (Table S5), LAB cautiously touches AM with its antennae and then abruptly withdraws in fear as type III behaviour.

In type IV behaviour (defence), AM rushed through the Petri dish, touching or colliding with LAB (Table S5). LAB raises its anterior legs distending the rostrum in a defensive position. On collision events, LAB may become stuck on AM by the anterior gluey legs. The reduviid moves backwards to break off the AM, stressing the anterior legs up, releasing them from AM seta. This interaction ended without AM pierced.

**Figure 18:**
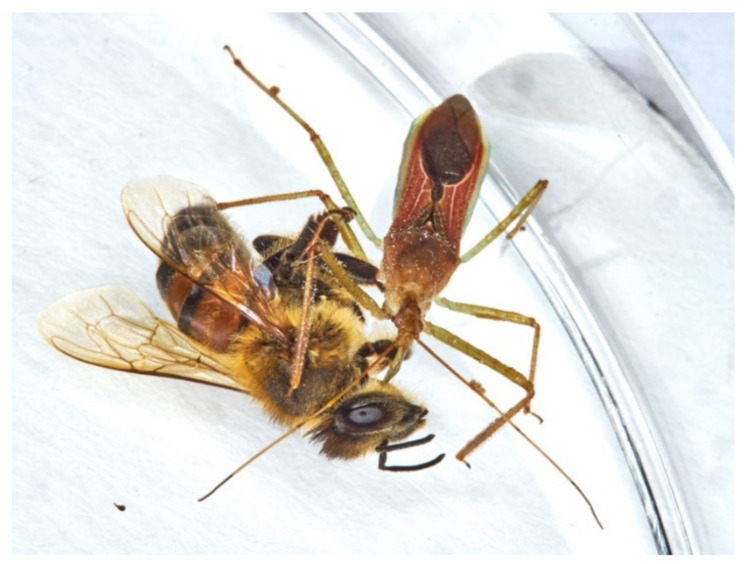
LAB female feeding on the single killed AM worker.

The test reveals that the 5% only of the forced interaction into a restricted arena led to one attack due to a defensive LAB need (Figure 18). Behaviours following the death of the honeybee, stylet insertion and use of the carcass are comparable with those of the other prey/carcasses.

The only predation resulted because of the sticking of AM on the LAB forelegs. Based on these observations, we suggest that AM is not among LAB regular prey.

**Figure 19:**
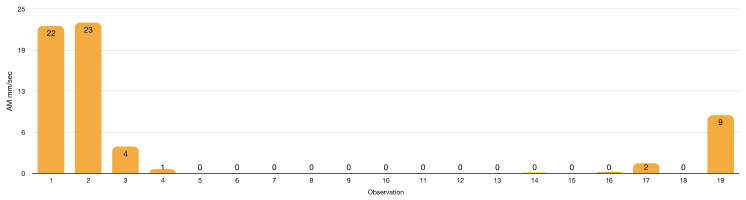
*Apis mellifera* speed in 9 cm Petri dish arena (details in Table S5).

**Figure 20:**
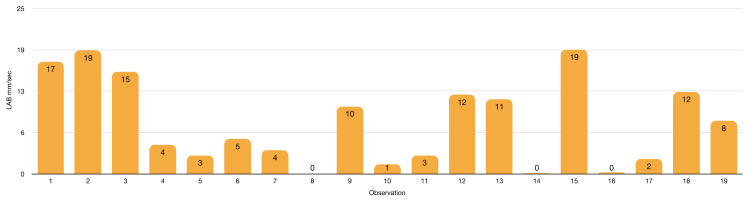
*Zelus renardii* speed in 9 cm Petri dish arena (details in Table S5).

Speed analysis (Figure 19 and Figure 20) of LAB–AM interactions can shed light on LAB predatory behaviour. LAB has shown ambushing capabilities with several preys. Therefore, the lack of LAB attacks of static AM strongly suggests a non-LAB prey status for this beneficial insect.

In the AM–LAB interactions, AM moves at an average speed of 3 mm/s (Table S5 and Figure 19) but often—68% of assays—it stays at the release spot. Little or no AMs motility could be related to the onset of starvation, which drives them to die 24–48 h after [191].

LABs show a faster average speed (8 mm/s) and mobility (578 mm) than AM during the interactions (Table S5 and Figure 20). Sex-related differences exist in *Z. renardii,* with females that are on average more mobile (943 mm) and faster (9 mm/s) than males. LAB females increased mobility could be related to a significant need for resources to perform vital functions, i.e., egg laying [192,193].

### 3.12. LAB and Humans

**Figure 21:**
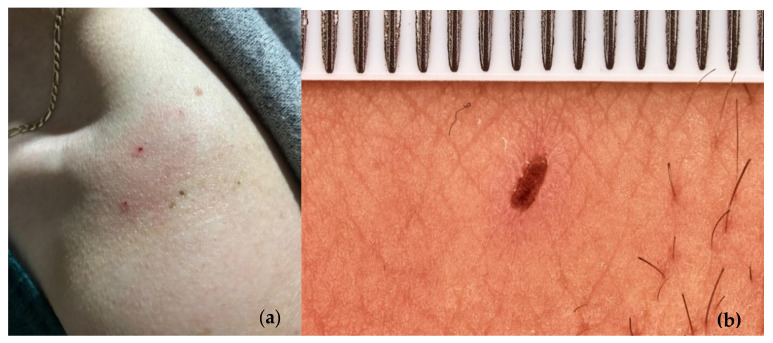
(**a**) Skin rashes on woman clavicle originated by overstressed LAB bite; (**b**) detail of skin-rush provoked by LAB biting on individual’s hand.

LABs bite humans occasionally but only when menaced [114,194,195]. LABs pierced the skin of eight out of ten volunteers. The piercings originate minor skin rashes a few mm in diameter (Figure 21). The pain is almost negligible and does not persist much after the wounding episode. LAB bites on humans are not frequent, and the consequences are similar to European *Polistes* spp. wasp stings on our experience. The day after the bite, the pain usually disappears, and only one weal was present with a culminant biting point and a 1–3 mm flat and non-infected scab. None of the volunteers reported any concerning clinical effects. The LABs involved in the tests died out from the interaction on the volunteers’ skin.

Reduviids produce a wide variety of toxins in their saliva that are possibly involved in skin reactions to piercing. The effects of the saliva toxins vary among vertebrates [196]. LAB bites may raise concern because some Reduviidae are well-known vectors of Chagas disease [197,198], however, this is not the case with LAB since it is not a hematophagous insect. Piercings are rare also because LAB bites for defence only, as reported for other Reduviidae [194,195]. The only possible risk could therefore be associated with allergic reactions in hypersensitive humans.

## 4. Conclusions

LAB is a Nearctic reduviid that entered the Mediterranean as an alien non-invasive species. This reduviid also acclimated in several circum-Mediterranean countries, roaming mainly in urban and peri-urban areas. The coexistence with humans seems more harmful for insects than humans since LAB provokes entomophobia reactions that often lead humans to kill it.

Gross opinions consider LAB a generalist predator, but evidence suggests that it is primarily stenophagous on Hemiptera. LAB chooses its prey according to their habitats, including the prey host plant, abundance, size and mobility of the species it encounters.

LAB was considered capable of disrupting natural biocontrol by intraguild predation. Still, this adverse side effect is probably due to oversimplified experiments in caging a restricted number of species and individuals in small environments.

*Zelus renardii* often seeks honeydew-contaminated plants because they may host potential prey. Honeydew could lure the reduviid on Hemiptera-infested plants provoking egg laying and subsequent LABs juvenile presence, eventually. Honeydew may aggregate the predator and persist in the place we need for pest control, consequently. Hopefully, future studies will isolate attractive semiochemicals for the manipulation of *Z. renardii* behaviour.

Nevertheless, not all honeydew-producing insects are prey for the *Z. renardii*, showing no interest in immotile instars of hemipteran. Tests confirm that LAB does not feed on Aleyrodidae, juveniles and immotile Pseudococcidae, Kermesidae, and Coccidae. LAB is an insectivore sometimes feeding on honeydews or plant secretions in prey absence, but without growing or laying eggs. The reduviid is not a plant pest or phytophagous.

All the LAB’s instars are cannibalistic in conditions of severe starvation that quickly occur in dense breeding. Adults can attack and kill naiades and nymphs, however, females may also kill males approaching for mating.

The distension of the reduviid ventral abdominal fold during feeding and the subsequent regular egg laying strongly suggest prey availability. Moreover, a prey that guarantees food enough for egg laying in tests may be functional for the predator, either in nature or the field.

Quantitative observations on *Z. renardii* can help us develop this predator as a biocontrol agent. LAB predation on *A. mellifera* and *H. axyridis* are rare events in nature and are also exceptional under laboratory conditions.

*Zelus renardii* can breed continuously with a constant supply of several living prey, namely *D. melanogaster*, *D. suzukii*, *M. scalaris*, *M. gladiata*, Aphrophoridae, Issidae, and Miridae. In rearing conditions, the predator reuses the carcasses of already killed prey.

Our tests confirm some Palearctic pests’ availability as prey, which is vicariant for the corresponding Nearctic species that have been well known for over a century of studies.

LAB displays different aggressiveness amongst the prey, behaving as stenophagous. LAB prefers prey able to meet its developmental and reproductive requirements. Some LAB attacks were driven by caging conditions as a defence reaction (i.e., honeybees). The tests suggest that *M. gladiata*, *P. spumarius*, *N. campestris*, *D. melanogaster, D. suzukii*, and *M. scalaris* are available pest prey for *Z. renardii*. Field experiments will further demonstrate the role of LAB as the olive fly (*B. oleae*) biocontrol agent. *D. melanogaster*, *D. suzukii*, and *M. scalaris* may help in *Z. renardii* mass or continuous rearing since those prey can be mass reared on artificial diets. *A. mellifera* does not appear as a prey for *Z. renardii*.

Experiments suggest that LAB is a biocontrol agent of some economically relevant olive trees pests. The LAB features analysed in this work make it a good candidate for inundative biocontrol programmes to manage *Xylella fastidiosa pauca* ST53 current pandemics, reducing the incidence of Xf new infections and transmissions. LAB can integrate olive IPM to mitigate damage by other olive-related pests, such as *B. oleae*.

## Figures and Tables

**Table 1:** *Zelus renardii* field survey in southern: Italy, Albania, and Spain.

Locations	Date	GPS	Plant Exploitation	Associate Insect
“2 Giugno” Public Park, Bari, Italy	24 September 2014	41.10250055° N; 16.8747697° E	*Quercus ilex* L., 1753	*Nidularia pulvinata* Planchon, 1864; *Kermes vermilio* Planchon, 1864
Campus “E. Quagliariello” University of Bari Aldo Moro, Italy	26 September 2014	41.103763° N; 16.859629° E	*Citrus* spp. L., 1753; *Pyracantha coccinea* M. Roem., 1847; *Pyrus communis* L., 1753; *Olea europaea* L., 1753; *Ficus carica* L., 1753	*Aleurocanthus spiniferus* (Quaintance, 1903); *Aleurothrixus floccosus* (Maskell, 1895); *Saissetia oleae* Olivier, 1791; *Homotoma ficus* (L., 1758)
“A. De Gasperi” Avenue 268, Bari, Italy	6 August 2015	41.104161° N; 16.871717° E	*Ailanthus altissima,* (Mill) Swingle; *Capsicum annuum* L., 1753; *Aloysia citriodora* Palau, 1784	*Aleurocanthus spiniferus* (Quaintance, 1903), *Aphis gossypii* Glover, 1877; *Aphis* spp. L., 1758
“Papa Pio XII” Avenue 32, Bari, Italy	21 August 2015	41.103763° N; 16.859629° E	*Ficus* spp. L., 1753	*Macrohomotoma gladiata* Kuwayama, 1908
“L. De Laurentis” Avenue, Bari, Italy	22 September 2015	41.093992° N; 16.864317° E	*Quercus ilex* L., 1753	*Nidularia pulvinata* Planchon, 1864; *Kermes vermilio* Planchon, 1864
S.S. 271, Km 9 Bari-Bitritto, Italy	15 April 2016	41.066386° N; 16.826862° E	*Olea europaea* L., 1753	*Saissetia oleae* Olivier, 1791
“G. Mazzini” Avenue, Valenzano, Italy	19 April 2016	41.04405° N; 16.88717° E	*Cupressus* spp. L.,1753	any significant insect presence
“Roma” Avenue, Copertino, Lecce, Italy	18 May 2016	40.268763° N; 18.050435° E	*Quercus ilex* L., 1753	*Nidularia pulvinata* Planchon, 1864; *Kermes vermilio* Planchon, 1864
“G. Toma” street 17, Bari, Italy	19 September 2017	41.108673° N; 16.872666° E	*Laurus nobilis* L.,1753	*Trioza alacris* Flor, 1861
Campus “E. Quagliariello” University of Bari Aldo Moro, Italy	20 September 2017	41.103763° N; 16.859629° E	*Laurus nobilis* L., 1753	*Trioza alacris* Flor, 1861; *Protopulvinaria pyriformis* (Cockerell, 1894)
“Azienda Martucci” farm University of Bari Aldo Moro, Valenzano, Bari, Italy	22 September 2017	41.022620° N; 16.904295° E	*Laurus nobilis* L., 1753	*Trioza alacris* Flor, 1861
“E. Montale” Street, Copertino, Lecce, Italy	3 March 2018	40.263238° N; 18.056953° E	*Laurus nobilis* L., 1753	*Trioza alacris* Flor, 1861
“Piazza dei Martiri” Gioia del Colle, Bari, Italy	29 September 2018	40.800365° N; 16.922870° E	*Chamaerops humilis* L., 1753	*Ommatissus binotatus* Fieber, 1875
“XXV Luglio” Avenue, Lecce, Italy	2 October 2018	40.353825° N; 18.173999° E	*Chamaerops humilis* L., 1753	*Ommatissus binotatus* Fieber, 1875
“Carrer Portugal” Alicante, Spain	15 September 2018	38.337504° N; 0.492043° E	*Oleae europaea* L., 1753	*Saissetia coffeae* (Walker, 1852)
“Demokracia” Street, Tirana, Albania	1 March 2019	41.3715552° N; 19.7776646° E	*Ocimum basilicum* L., 1753	any significant insect presence
“A. Jashari” Street, Laknas, Albania	4 March 2019	41.3687091° N; 19.7426623° E	*Oleae europaea* L., 1753	any significant insect presence

**Table 2:** *Zelus* predation test arenas details.

Prey Species	Prey Taxa	Cage Area	Cage Volume	Prey Density	Notes
*Macrohomotoma gladiata*	(Hemiptera: Psyllidae)	9.18 × 7.12 × 8.9 cm	Approx. 580 cm^3^	>0.06 prey/cm^3^	Nymphs aggregate on deformed twigs. Adults are vagrant.
*Planococcus citri*	(Hemiptera: Pseudococcidae)	8.55 cm (∅); 1.42 cm (h)	81 cm^3^	0.06 prey/cm^3^	*Zelus* only preys on moving individuals
*Planococcus ficus*	(Hemiptera: Pseudococcidae)	8.55 cm (∅); 1.42 cm (h)	81 cm^3^	0.06 prey/cm^3^	*Zelus* only preys on moving individuals
*Aleurothrixus floccosus*	(Hemiptera: Aleyrodidae)	9.18 × 7.12 × 8.9 cm	Approx. 580 cm^3^	*circa* 0.6 prey/cm^3^	Approx. 50 individuals per leaf
*Aleurocanthus spiniferus*	(Hemiptera: Aleyrodidae)	9.18 × 7.12 × 8.9 cm	Approx. 580 cm^3^	*circa* 0.6 prey/cm^3^	Approx. 50 individuals per leaf
*Latilica* sp.	(Hemiptera: Issidae)	8.55 cm (∅); 1.42 cm (h)	81 cm^3^	0.01 prey/cm^3^	In mixed prey ensemble
*Kermes vermilio*	(Hemiptera: Kermesidae)	9.18 × 7.12 × 8.9 cm	Approx. 580 cm^3^	min. 1.2 prey/cm^3^	2–3 adult females and hundreds of crawlers
*Nidularia pulvinata*	(Hemiptera: Kermesidae)	9.18 × 7.12 × 8.9 cm	Approx. 580 cm^3^	min. 1.2 prey/cm^3^	2–3 adult females, hundreds of crawlers
*Saissetia oleae*	(Hemiptera: Coccidae)	9.18 × 7.12 × 8.9 cm	Approx. 580 cm^3^	From 0.02 to 0.03 prey/cm^3^	2–3 nymphs underside each olive leaf
*Harmonia axyridis*	(Coleoptera: Coccinellidae)	8.55 cm (∅); 1.42 cm (h)	81 cm^3^	0.01 prey/cm^3^	LAB preys the ladybeetle without killing it
*Apis mellifera*	(Hymenoptera: Apidae)	8.55 cm (∅); 1.42 cm (h)	81 cm^3^	0.01 prey/cm^3^	Workers
*Philaenus spumarius*	(Hemiptera: Aphrophoridae)	10 × 10 × 5 cm	Approx. 500 cm^3^	0.02 prey/cm^3^	As in [11]
*Neophilaenus campestris*	(Hemiptera: Aphrophoridae)	10 × 10 × 5 cm	approx. 500 cm^3^	0.02 prey/cm^3^	As in [11]
*Philaenus spumarius*	(Hemiptera: Aphrophoridae)	Falcon	60 cm^3^	from 0.15 to 0.25 prey/cm^3^	In mixed prey ensemble
*Neophilaenus campestris*	(Hemiptera: Aphrophoridae)	Falcon	60 cm^3^	from 0.15 to 0.25 prey/cm^3^	In mixed prey ensemble
Issidae	(Hemiptera)	Falcon	60 cm^3^	from 0.15 to 0.25 prey/cm^3^	In mixed prey ensemble
Miridae	(Hemiptera)	Falcon	60 cm^3^	from 0.15 to 0.25 prey/cm^3^	In mixed prey ensemble
*Bactrocera oleae*	(Diptera: Tephritidae)	9.18 × 7.12 × 8.9 cm	Approx. 580 cm^3^	0.03 prey/cm^3^	LAB preys the olive fly

**Table 3:** *Zelus renardii* presence in plant and associated honeydew-producing Hemipteran pests. Data refer to Table 1. Abbreviations: N1–N5 = juvenile instars; Ad = adult.

Host Plant	Associated Hemipteran	LAB Instars						
		Egg	N1	N2	N3	N4	N5	Ad
*Quercus ilex*	*Nidularia pulvinata*							4
*Quercus ilex*	*Kermes vermilio*							1
*Olea europaea*	*Saissetia oleae*							1
*Olea europaea*	*Saissetia coffeae*							2
*Ficus microcarpa*	*Macrohomotoma gladiata*					1		1
*Ficus carica*	*Homotoma ficus*	2				1		4
*Ailanthus altissima*	*Aleurocanthus spiniferus*	7		1	2			1
*Pyracantha coccinea*	*Aleurocanthus spiniferus*			1	1	2		5
*Pyrus communis*	*Aleurocanthus spiniferus*							14
*Citrus CV*	*Aleurocanthus spiniferus*		1			2	3	18
*Citrus CV*	*Aleurothrixus floccosus*	12				1	3	4
*Aloysia citriodora*	*Aphis* spp.						1	1
*Capsicum annuum*	*Aphis* spp.							2
*Chamaerops humilis*	*Ommatissus binotatus*			6	1		2	5
*Laurus nobilis*	*Trioza alacris*	11		1				1

## Data Availability

The authors can share the references used to write this contribution.

## Supplementary Materials

The following are available online at www.mdpi.com/article/10.3390/insects13020158/s1, Table S1: *Zelus renardii* prey species preferences with order and family, the corresponding reference with the types of approach and experiments. Table S2: Raw data regarding *Zelus renardii* mating. The data consider: “Date” of mating; “Mating time (min)” is the time in minutes spent for each couple to complete the mating; “Days After Mating (d)” represents the days spent between mating and egg laying; “Eggs Laid” represents the total number of eggs laid; “Eggs Hatched” shows the total numbers of eggs hatched; “Embryonic Development (d)” shows the days spent between eggs laying and eggs hatching. *C* = couple. Data graphed Figure 7. Table S3: Raw data regarding *Zelus renardii* preying Aphrophoridae, Miridae and Issidae assemblage. The data consider: “Date” of predation; “Place” considers the locations where trials occurred; “N” shows the progressive number used to identify the predator; “M-F” reports the sexing of predators; “Prey 01–15” reports the progressive number of prey; “Time Sum” accumulates the minutes’ employees by each predator to feed all prey; “Prey n” reports the total numbers of prey preyed. *M* = Male; *F* = Female and *Dept.* = Laboratory of Department. Data graphed Figure 11 and Figure 12. Table S4: Raw data regarding *Zelus renardii* preying *Bactrocera oleae*. The data consider: “Date” of predation; “Place” considers the trials locations; “N” shows the progressive number used to identify the predator; “SEX” reports the sexing of predators; “P 01–12” reports the progressive number of *B. oleae* tested; “Time Sum” accumulates the minutes’ employees by each predator to feed all prey; “Prey n” reports the total numbers of prey preyed. *M* = Male; *F* = Female and *Dept.* = Laboratory of Department. Data graphed Figure 15 and Figure 16. Table S5: Raw data regarding *Zelus renardii* and *Apis mellifera* interaction. The “N” shows the progressive number used to identify the interaction between *Z. renardii* and *A. mellifera;* “Interactions Sec” reports the time in seconds of each interaction; “AM track (mm)” shows the distance travelled by *A. mellifera* in the arena expressed in millimetres; “ZR track (mm)” shows the distance travelled by *Z. renardii* in the arena noted in millimetres; “AM mm/sec” reports the average speed of the *A. mellifera* during interaction with the predator; “AM mm/sec” reports the average speed of the *Z. renardii* during interaction with the pollinator; “Interaction” notes the behaviour of *Z. renardii* and *A. mellifera* during trials. Data graphed Figure 19 and Figure 20. *Zelus*-*Apis* Movie: The movie shows interaction n.19 (Figure 17) between *Z. renardii* and *A. mellifera*. Initially, *A. mellifera* and *Z. renardii* stick on *Zelus* anterior legs. Later, the fluttering honeybee accidentally bumps *Z. renardii*, eliciting its defence biting reaction that will kill the honeybee. Later, the *Zelus* feed on dead honeybees (Figure 18) as on other dead insects.
