# Supplementary material for: Zelus renardii Roaming in Southern Italy
Source: Insects. 2022 Jan 31;13(2):158. doi: 10.3390/insects13020158 (PMC8878308; doi:10.3390/insects13020158)
Supplement: Supplementary file 1 [file insects-13-00158-s001.zip › insects-1458095-supplementary/Table S1.pdf]

Order & Family	Species	Reference	Approach	Experiments
Blatt. Blattidae	*Periplaneta americana* (L., 1758)	[51]	Qualitative	Lab
Orth. Gryllidae	*Acheta domesticus* (L., 1758)	[51]	Qualitative	Lab
Thys. Thripidae	*Scirtothrips citri* (Moulton, 1909)	[52]	Qualitative	Lab
		[53]	Qualitative	Lab
	Scirtothrips perseae Nakahara, 1997	[54]	Qualitative	Field
	Frankliniella occidentalis Pergande, 1895	[54]	Qualitative	Field
	Frankliniella insularis Franklin, 1908	[54]	Qualitative	Field
Hem. Pseudococcidae	*Planococcus citri* (Risso, 1813)	[53]	Qualitative	Lab
	Pseudococcus citriculus Green, 1922	[55]	Qualitative	Lab
	Ferrisia gilli Gullan, 2003	[56]	Qualitative	Field
Hem. Eriococcidae	*Acanthococcus lagerstroemiae* (Kuwana, 1907)	[57]	Qualitative	Lab
Hem. Psyllidae	*Glycaspis brimblecombei* Moore, 1964	[58]	Qualitative	Field
		[59]	Qualitative	Field
	Macrohomotoma gladiata Kuwayama, 1908	[6]	Quantitative	Field
	Heteropsylla cubana Crawford, 1914	[60]	Qualitative	Field
		[61]	Qualitative	Field
Hem. Liviidae	*Diaphorina citri* Kuwayama, 1908	[62]	Qualitative	Field
		[63]	Quantitative	Field
		[64]	Quantitative	Lab
		[65]	Qualitative	Field
		[66]	Quantitative	Field
Hem. Aleyrodidae	*Bemisia tabaci* (Gennadius, 1889)	[67]	Qualitative	Field
		[68]	Quantitative	Field
		[69]	Quantitative	Field
		[70]	Quantitative	Field
Hem. Aphididae	*Therioaphis maculata* (Buckton, 1899)	[71]	Qualitative	Field
	Aphis gossypii Glover, 1877	[15]	Qualitative	Field
		[16]	Quantitative	Field
		[17]	Quantitative	Field
		[72]	Quantitative	Lab
		[73]	Quantitative	Field
		[74]	Quantitative	Field
		[75]	Quantitative	Field
	Brachycaudus tragopogonis (Kalt., 1843)	[62]	Qualitative	Field
		[76]	Quantitative	Lab
	Brachycaudus persicae (Passerini, 1860)	[76]	Quantitative	Lab
	Macrosiphum pisi (Harris, 1776)	[77]	Qualitative	Lab
	Macrosiphum rosae (L., 1758)	[62]	Qualitative	Field
		[76]	Quantitative	Lab
	Macrosiphum euphorbiae L., 1758	[76]	Quantitative	Lab
	Myzus persicae (Sulzer, 1776)	[62]	Qualitative	Field
		[76]	Quantitative	Lab
	Therioaphis maculata (Buckton, 1899)	[71]	Qualitative	Field
Hem. Cicadellidae	*Carneocephala* spp.	[77]	Quantitative	Lab
	Draeculacephala spp.	[77]	Quantitative	Lab
	Acinopterus spp.	[77]	Quantitative	Lab
	Aceratagalia spp.	[77]	Quantitative	Lab
	Homalodisca vitripennis (Germar, 1821)	[78]	Quantitative	Lab
		[79]	Quantitative	Lab
		[80]	Quantitative	Lab
Hem. Aphrophoridae	*Philaenus spumarius* L., 1758	[81]	Quantitative	Lab
Hem. Membracidae	*Spissistilus festinus* (Say, 1866)	[82]	Qualitative	Field
Hem. Dictyopharidae	*Dictyophara europaea* (L., 1767)	[51]	Qualitative	Lab
Hem. Delphacidae	*Perkinsiella saccharicida* Kirkaldy, 1903	[83]	Qualitative	Field
		[84]	Qualitative	Field
		[85]	Qualitative	Field
		[86]	Qualitative	Field
		[87]	Qualitative	Field
	Peregrinus maidis (Ashmead, 1890)	[88]	Qualitative	Field
		[89]	Qualitative	Field
		[90]	Qualitative	Field
		[91]	Qualitative	Field
Hem. Anthocoridae	*Orius tristicolor* (White, 1879)	[17]	Quantitative	Field

Hem. Miridae	*Pycnoderes quadrimaculatus* Guérin-Méneville, 1857	[90]	Qualitative	Field
		[92]	Qualitative	Field
	Lygus spp.	[77]	Qualitative	Field
	Lygus hesperus (Knight, 1917)	[17]	Quantitative	Field
		[69]	Quantitative	Field
		[70]	Quantitative	Field
		[72]	Quantitative	Lab
		[93]	Quantitative	Field
		[94]	Quantitative	Lab
		[95]	Qualitative	Field
		[96]	Quantitative	Field
Hem. Rhopalidae	*Liorhyssus hyalinus* (Fabricius, 1794)	[90]	Qualitative	Field
Hem. Geocoridae	*Geocoris* spp.	[97]	Quantitative	Field
	Geocoris punctipes (Say, 1832)	[70]	Quantitative	Field
	Geocoris pallens Stål, 1854	[17]	Quantitative	Field
		[18]	Quantitative	Field
Hem. Plataspidae	*Megacopta cribraria* (Fabricius, 1798)	[98]	Quantitative	Field
		[99]	Qualitative	Field
		[100]	Quantitative	Field
Hem. Pentatomidae	*Chlorochroa ligata* (Say, 1832)	[101]	Qualitative	Lab
	Euschistus servus (Say, 1832)	[102]	Quantitative	Lab
		[103]	Quantitative	Lab
	Euschistus tristigmus (Say, 1832)	[102]	Quantitative	Lab
	Euschistus impictiventris Stål, 1872	[104]	Qualitative	Lab
	Nezara viridula L., 1758	[51]	Qualitative	Lab
		[102]	Quantitative	Lab
		[105]	Quantitative	Lab
	Eurydema spp.	[51]	Qualitative	Lab
Neur. Chrysopidae	*Chrysoperla carnea* Stephens, 1836	[15]	Quantitative	Field
		[16]	Quantitative	Field
		[55]	Qualitative	Field
		[106]	Qualitative	Field
		[107]	Qualitative	Field
		[108]	Qualitative	Field
		[109]	Quantitative	Field
		[110]	Quantitative	Lab
		[111]	Quantitative	Lab
	Chrysoperla rufilabris (Burmeister, 1839)	[108]	Qualitative	Field
	Chrysopa nigricornis Burmeister, 1839	[15]	Quantitative	Field
		[108]	Quantitative	Field
	Chrysopa oculata Say, 1839	[108]	Qualitative	Field
	Chrysopa coloradensis Banks, 1895	[108]	Qualitative	Field
Lep. Limacodidae	*Darna trima* Moore, 1859	[112]	Qualitative	Field
Lep. Bucculatricidae	*Bucculatrix thurberiella* Busck, 1914	[72]	Quantitative	Lab
		[86]	Quantitative	Lab
		[113]	Qualitative	Lab
Lep. Gelechiidae	*Pectinophora gossypiella* (Saunders, 1844)	[67]	Quantitative	Lab
		[72]	Quantitative	Lab
		[114]	Qualitative	Lab
		[115]	Quantitative	Field
Lep. Tortricidae	*Platynota stultana* Walsingham, 1884	[116]	Qualitative	Field
Lep. Papilionidae	*Papilio zolocaon* Boisduval	[117]	Qualitative	Field
Lep. Pieridae	*Colias eurytheme* Boisduval, 1852	[77]	Qualitative	Lab
Lep. Noctuidae	*Helicoverpa armigera* (Hübner, 1808)	[118]	Qualitative	Field
	Helicoverpa zea (Boddie, 1850)	[72]	Quantitative	Lab
		[119]	Quantitative	Field
		[120]	Quantitative	Lab
		[121]	Quantitative	Lab
		[122]	Quantitative	Lab
	Heliothis virescens (Fabricius, 1777)	[120]	Quantitative	Lab
		[121]	Quantitative	Lab
		[123]	Qualitative	Field
		[124]	Quantitative	Lab
		[125]	Quantitative	Lab
	Spodoptera exigua (Hübner, 1808)	[120]	Quantitative	Lab

		[121]	Quantitative	Lab
		[125]	Quantitative	Lab
		[126]	Quantitative	Field
		[127]	Quantitative	Lab
	Spodoptera praefica (Grote, 1875)	[128]	Qualitative	Field
	Spodoptera frugiperda (Smith, 1797)	[129]	Quantitative	Lab
	Trichoplusia ni (Hübner, 1800–1803)	[72]	Quantitative	Lab
		[129]	Quantitative	Lab
Lep. Geometridae	*Anacamptodes fragilaria* Grossbeck, 1909	[130]	Qualitative	Field
Dip. Drosophilidae	*Drosophila melanogaster* Meigen,1830	[62]	Qualitative	Lab
		[77]	Qualitative	Lab
		[131]	Qualitative	Lab
Dip. Tephritidae	*Dacus cucurbitae* Coquillett, 1849	[84]	Qualitative	Field
		[132]	Qualitative	Field
	Anastrepha ludens Loew, 1873	[62]	Qualitative	Lab
	Ceratitis capitata Wiedemann, 1824	[63]	Qualitative	Lab
Col. Coccinellidae	*Adalia bipunctata* (L., 1758)	[62]	Qualitative	Field
		[76]	Quantitative	Lab
	Cryptolaemus montrouzieri Mulsant, 1850	[62]	Qualitative	Field
		[76]	Quantitative	Lab
	Eriopis connexa (Germar,1824)	[62]	Qualitative	Field
		[76]	Quantitative	Lab
	Hippodamia variegata (Goeze, 1777)	[62]	Qualitative	Field
		[76]	Quantitative	Lab
	Harmonia axyridis (Pallas, 1773)	[133]	Qualitative	Field
Col. Chrysomelidae	*Mimosestes sallaei* Sharp, 1885	[134]	Qualitative	Field
	Bruchus rufimanus Boheman, 1833	[135]	Qualitative	Field
		[136]	Quantitative	Field
		[137]	Qualitative	Field
	Diorhabda elongate Brullé, 1832	[138]	Qualitative	Field
	Diabrotica undecimpunctata Mannerheim, 1843	[139]	Qualitative	Field
Col. Curculionidae	*Anthonomus grandis grandis* Boheman, 1843	[140]	Qualitative	Field
Col. Melyridae	*Collops vittatus* (Say, 1823)	[70]	Quantitative	Field
Hym. Scoliidae	*Campsomeris marginella modesta* Smith, 1855	[141]	Qualitative	Field
	Scolia manilae (Ashmead, 1885)	[141]	Qualitative	Field
Hym. Apidae	*Apis mellifera* L., 1758	[84]	Qualitative	Field
		[141]	Qualitative	Field
Hym. Aphelinidae	*Aphytis* spp.	[142]	Qualitative	Field
	Aphytis aonidiae Mercet, 1911	[143]	Quantitative	Field

Zelus renardii prey species preferences with order and family, the corresponding reference with the types of approach and experiments.
