# Supplementary material for: Zelus renardii Roaming in Southern Italy
Source: Insects. 2022 Jan 31;13(2):158. doi: 10.3390/insects13020158 (PMC8878308; doi:10.3390/insects13020158)
Supplement: Supplementary file 1 [file insects-13-00158-s001.zip › insects-1458095-supplementary/Table S2.pdf]

Egg-Batch	Date	Mating time (min)	Days after mating (d)	Eggs laid	Eggs hacthed	Embryonic development (d)
**EB01**	09/04/17	38	10	21	21	12
**EB02**	06/08/17	35	66	32	32	13
**EB03**	28/08/17	29	76	29	26	20
**EB04**	30/08/17	31	6	22	3	13
**EB05**	31/08/17	31	52	23	14	27
**EB06**	01/09/17	32	70	38	32	23
**EB07**	04/09/17	34	137	21	16	13
**EB08**	06/09/17	35	70	29	26	21
**EB09**	06/09/17	32	83	30	28	23
**EB10**	06/09/17	34	71	34	32	11
**EB11**	12/09/17	38	105	19	7	18
**EB12**	11/10/17	34	123	26	6	9
**EB13**	18/10/17	42	91	29	27	23
**EB14**	08/11/17	43	124	30	30	9
**EB15**	08/11/17	45	132	32	22	14
**EB16**	08/11/17	45	139	20	18	13
**EB17**	09/11/17	42	125	21	21	16
**EB18**	09/11/17	36	83	13	11	17
**EB19**	09/11/17	41	96	30	3	22
**EB20**	16/11/17	39	143	35	33	16
**EB21**	17/11/17	38	38	21	13	29
**EB22**	17/11/17	31	57	36	36	20
**EB23**	18/11/17	36	63	21	20	22
**EB24**	18/11/17	34	72	17	10	25
**EB25**	07/03/18	54	32	11	11	21
**EB26**	08/03/18	34	90	36	31	17
**EB27**	09/04/18	47	39	23	22	17
**EB28**	09/04/18	37	29	31	28	18
**EB29**	03/06/18	36	53	48	48	12
**EB30**	04/06/18	29	57	27	26	8

The table shows the raw data about *Zelus renardii* mating. The data considers "Date" of mating; the "Mating time (min)" is the time in minutes spent for each couple to complete the mating; the "Days After Mating (d)" represent the days spent between mating and egg-laying; "Eggs Laid" represent the total number of eggs laid; the "Eggs Hatched" shows the total numbers of eggs hatched; the "Embryonic Development (d)" shows the days spent between eggs laying and eggs hatching. *EB*= Egg-Batch. Data graphed the Fig. 7.
