# Supplementary material for: Zelus renardii Roaming in Southern Italy
Source: Insects. 2022 Jan 31;13(2):158. doi: 10.3390/insects13020158 (PMC8878308; doi:10.3390/insects13020158)
Supplement: Supplementary file 1 [file insects-13-00158-s001.zip › insects-1458095-supplementary/Table S3.pdf]

Data	Place	N	M-F	Prey 01	Prey 02	Prey 03	Prey 04	Prey 05	Prey 06	Prey 07	Prey 08	Prey 09	Prey 10	Prey 11	Prey 12	Prey 13	Prey 14	Prey 15	Time sum	Prey n
**2016 10 01**	**Home**	**1**	**M**	105	47	42	90	60	41	34	20	9	8	11	1	2	1	2	473	15
**2016 10 01**	**Dept.**	**2**	**M**	66	65	61	58	48	76	28	28	6	17	13	9	13	8	8	504	15
**2016 10 03**	**Dept.**	**3**	**F**	73	68	59	62	58	77	43	22	12	10	9	12				505	12
**2016 10 03**	**Dept.**	**4**	**F**	89	68	53	47	64	87	38	23	7	13	12	6	14	3		524	14
**2016 10 03**	**Dept.**	**5**	**F**	71	51	58	48	68	43	56	25	8	22	15	10	12			487	13
**2016 10 04**	**Dept.**	**6**	**M**	70	65	62	49	39	79	76	23	4	11	15	3	2			498	13
**2016 10 04**	**Dept.**	**7**	**M**	73	61	65	56	21	89	49	18	3	10	12	9	3	2	5	476	15
**2016 10 04**	**Dept.**	**8**	**M**	65	47	49	63	78	62	39	24	8	15	13	11	11	4		489	14
**2016 10 05**	**Dept.**	**9**	**M**	58	40	38	29	44	57	25	21	12	11	15					350	11
**2016 10 11**	**Dept.**	**10**	**F**	54	48	41	87	39	71	19	14	6	13						392	10
**2016 10 11**	**Dept.**	**11**	**M**	74	57	52	43	76	62	28	18	12	13	14	6	6	8	2	471	15
**2016 10 13**	**Dept.**	**12**	**M**	79	65	60	55	34	60	21	19	15	16	13	7				444	12
**2016 10 13**	**Dept.**	**13**	**M**	67	53	61	39	75	67	44	20	11	14	11	15	9	10		496	14
**2016 10 18**	**Dept.**	**14**	**M**	59	54	47	63	48	59	29	13	17	12	14	10	3	13	4	445	15
**2016 10 22**	**Home**	**15**	**F**	72	59	88	60	59	55	86	22	14	13						528	10
**2016 10 22**	**Home**	**16**	**M**	74	53	33	82	43	39	51	23	16	7	9	2	4	13	5	454	15
**2016 10 24**	**Dept.**	**17**	**F**	69	68	53	72	91	60	37	45	11	14	11	4	8	14		557	14
**2016 10 25**	**Dept.**	**18**	**M**	98	54	56	102	75	60	41	42	17	13	16	11	8	12	12	617	15
**2016 10 25**	**Dept.**	**19**	**M**	90	49	46	72	56	44	25	24	22	3	12	9	5	15	3	475	15
**2016 10 27**	**Dept.**	**20**	**F**	64	69	69	51	73	67	22	24	11							450	9
**2016 10 29**	**Home**	**21**	**F**	69	45	79	58	51	87	54	25								468	8

The table shows the raw data about *Zelus renardii* preying Aphrophoridae, Miridae and Issidae assemblage. The data considers "Date" of predation; the "Place" considers the locations where trials were conducted; the "N" shows the progressive number used to identify the predator; the "M-F" reports the sexing of predators; the "Prey 01–15" reports the progressive number of prey; the "Time Sum" accumulates the minutes' employees by each predator to feed all prey; the "Prey n" reports the total numbers of prey preyed. *M*= Male; *F*= Female and *Dept.* = Laboratory of Department. Data graphed the Fig.s 11 and 12.
