# Supplementary material for: Zelus renardii Roaming in Southern Italy
Source: Insects. 2022 Jan 31;13(2):158. doi: 10.3390/insects13020158 (PMC8878308; doi:10.3390/insects13020158)
Supplement: Supplementary file 1 [file insects-13-00158-s001.zip › insects-1458095-supplementary/Table S4.pdf]

Data	Place	N	SEX	P01	P02	P03	P04	P05	P06	P07	P08	P09	P10	P11	P12	Time sum	Pn
**2020 11 22**	**Dept.**	**1**	**M**	71	55	57	41	36	36	30	28	21	21	20		416	11
**2020 11 22**	**Dept.**	**2**	**M**	63	51	49	42	44	39	32	33	26	23	19	19	440	12
**2020 11 22**	**Dept.**	**3**	**M**	61	55	66	57	45	37	34	35	30	24	20		464	11
**2020 11 22**	**Dept.**	**4**	**M**	59	62	59	44	41	34	34	28	26	23	21	15	446	12
**2020 11 22**	**Dept.**	**5**	**M**	45	49	56	49	31	39	37	29	27				362	9
**2020 11 22**	**Dept.**	**6**	**M**	62	56	45	50	39	34	38	29	28	25	23		429	11
**2020 11 22**	**Dept.**	**7**	**M**	50	58	58	51	49	34	36	22	21	22	19	14	434	12
**2020 11 22**	**Dept.**	**8**	**M**	56	52	57	45	46	43	44	28	22	27	21	20	461	12
**2020 11 22**	**Dept.**	**9**	**M**	66	61	56	32	44	35	36	34	20	21	19	23	447	12
**2020 11 22**	**Dept.**	**10**	**M**	72	54	66	45	43	40	44	29	35	20	18	16	482	12
**2020 11 22**	**Dept.**	**11**	**M**	58	47	55	46	34	34	42	35	25	29			405	10

The table shows the raw data about *Zelus renardii* preying *Bactrocera oleae*. The data considers "Date" of predation; the "Place" considers the locations where trials were conducted; the "N" shows the progressive number used to identify the predator; the "SEX" reports the sexing of predators; the "P 01–12" reports the progressive number of *B. oleae* tested; the "Time Sum" accumulates the minutes' employees by each predator to feed all prey; the "Prey n" reports the total numbers of prey preyed. *M*= Male; *F*= Female and *Dept.* = Laboratory of Department. Data graphed the Fig.s 15 and 16.
