# Supplementary material for: Zelus renardii Roaming in Southern Italy
Source: Insects. 2022 Jan 31;13(2):158. doi: 10.3390/insects13020158 (PMC8878308; doi:10.3390/insects13020158)
Supplement: Supplementary file 1 [file insects-13-00158-s001.zip › insects-1458095-supplementary/Table S5.pdf]

Tabella 1

N	Interactions Sec	AM track (mm)	ZR track (mm)	AM mm/sec	ZR mm/sec	Interaction
1	43s	965	729	22	17	1 - reciprocal ignorance, wandering around in the Petri
2	45s	1032	838	23	19	1 - reciprocal ignorance, wandering around in the Petri
3	46s	187	708	4	15	1 - reciprocal ignorance, wandering around in the Petri
4	118s	76	515	1	4	1 - reciprocal ignorance, wandering around in the Petri
5	36s	0	100	0	3	1 - reciprocal ignorance, wandering around in the Petri
6	31s	0	163	0	5	1 - reciprocal ignorance, wandering around in the Petri
7	12s	0	43	0	4	1 - reciprocal ignorance, wandering around in the Petri
8	30s	0	0	0	0	2 - staying almost immotile near or on the release spot
9	387s	0	3947	0	10	1 - reciprocal ignorance, wandering around in the Petri
10	39s	0	56	0	1	1 - reciprocal ignorance, wandering around in the Petri
11	56s	0	158	0	3	1 - reciprocal ignorance, wandering around in the Petri
12	123s	0	1469	0	12	1 - reciprocal ignorance, wandering around in the Petri
13	58s	0	654	0	11	1 - reciprocal ignorance, wandering around in the Petri
14	2251s	308	293	0	0	5 - AM glues on LAB sticking legs that attacks and kills the honeybee defencing itself
15	5s	0	94	0	19	1 - reciprocal ignorance, wandering around in the Petri
16	124s	30	27	0	0	1 - reciprocal ignorance, wandering around in the Petri
17	26s	41	59	2	2	1 - reciprocal ignorance, wandering around in the Petri
18	6s	0	74	0	12	1 - reciprocal ignorance, wandering around in the Petri
19	133s	1182	1065	9	8	3 - AM walks or flutters versus the escaping LAB; 4 - AM accidentally collide with LAB eliciting its aggressive posture

The table shows the raw data about *Zelus renardii* and *Apis mellifera* interaction. The "N" shows the progressive number used to identify the interac2on between *Z. renardii* and *A. mellifera;* the "Interactions Sec" reports the 2me in seconds of each interaction; the "AM track (mm)" shows the distance travelled by *A. mellifera* in the arena expressed in millimetres; the "ZR track (mm)" shows the distance travelled by *Z. renardii* in the arena noted in millimetres; the "AM mm/sec" reports the average speed of the *A. mellifera* during interaction with the predator; the "AM mm/sec" reports the average speed of the *Z. renardii* during interaction with the pollinator; the "Interaction" notes the behaviour of *Z. renardii* and *A. mellifera* during trials. Data graphed the Fig.s 19 and 20.
